# Supplementary material for: Get Set or Get Distracted? Disentangling Content-Priming and Attention-Catching Effects of Background Lure Stimuli on Identifying Targets in Two Simultaneously Presented Series
Source: Brain Sci. 2019 Dec 11;9(12):365. doi: 10.3390/brainsci9120365 (PMC6955916; doi:10.3390/brainsci9120365)
Supplement: Supplementary file 1 [file brainsci-09-00365-s001.pdf]

Table S1. Percentages of trials with correctly identified targets.

	Target: Left			Target: Right		
T1–T2 Lag	1	3	3	1	3	3
T1&T2 Side	diff	same	diff	diff	same	diff
			**T1**			
no lures	80.9 (10.2)	85.1 (11.7)	82.6 (11.4)	83.3 (10.8)	89.9 (10.7)	80.9 (10.3)
digit lures, same side as T2	77.0 (12.3)	82.2 (16.3)	82.0 (13.0)	85.2 (12.5)	79.9 (10.6)	89.3 (8.0)
digit lures, other side than T2	84.7 (10.2)	83.3 (11.3)	82.0 (13.9)	83.1 (12.8)	87.2 (10.7)	83.5 (15.1)
color lures, same side as T2	82.8 (14.6)	84.3 (14.3)	84.8 (12.3)	81.8 (11.8)	76.8 (14.9)	80.6 (12.9)
color lures, other side than T2	81.7 (16.0)	76.8 (19.1)	81.1 (11.0)	81.6 (12.5)	81.0 (14.3)	82.0 (14.0)
			**T2**			
no lures	57.8 (24.3)	75.3 (15.0)	66.5 (19.7)	46.8 (22.7)	67.5 (20.3)	53.5 (23.8)
digit lures, same side as T2	55.9 (20.9)	60.4 (19.5)	65.9 (17.7)	47.9 (18.4)	64.3 (18.0)	64.2 (18.3)
digit lures, other side than T2	65.8 (16.3)	79.8 (16.4)	72.6 (19.9)	48.0 (23.0)	64.9 (12.1)	58.1 (24.5)
color lures, same side as T2	52.7 (28.3)	63.4 (16.2)	66.7 (16.8)	43.6 (17.0)	62.9 (12.8)	51.1 (14.0)
color lures, other side than T2	54.0 (22.2)	60.0 (16.2)	63.1 (23.5)	42.6 (26.2)	63.2 (18.0)	44.3 (20.4)

The upper half shows T1 values, the lower half T2 values. Values are mean percentages (and standard deviations) across participants. Numbers of trials with correctly identified targets were referred to all trials, both for T1 and T2 (rather than referring T2 to only those trials in which T1 was identified) to have the same scale for lure effects on T1 and on T2.
